# Report of a Case of Aneurism of the Ascending Aorta, Involving the Innominate Secondarily, Operated upon by Simultaneous Ligation of the Common Carotid and Subclavian-Cure

**Published:** 1889-01

**Authors:** F. T. Meriwether

**Affiliations:** Surgeon Mission Hospital, Asheville, N. C.


### REPORT OF A CASE OF ANEURISM OF THE ASCENDING AORTA, INVOLVING THE INNOMINATE SECONDARILY, OPERATED UPON BY SIMULTANEOUS LIGATION OF THE COMMON CAROTID AND SUBCLAVIAN-CURE.*

BY F. F. MERIWETHER, M. D.,

Surgeon Mission Hospital, Asheville, N. C.

Oct. 2[illegible]th, 1888.—Found patient, Mrs. P., aged 37 years, married, multipara, last child about three years of age. No history of syphilis as far back as third generation. The patient's two aunts, two uncles and grandmother on her mother's side, had died suddenly from some heart lesion, but she did not know exact trouble. Her father had died from acute tuberculosis. Patient had had a slight cough for some time and for last few weeks had suffered from asthma at night. For past six or eight months had been much annoyed by a buzzing and humming in her right ear, and for past few weeks had noticed a stiffness and some tenderness in right cervical region. Had had more or less pain in upper thorax for a year or more but on Friday, (Oct. 19, '88,) upon walking up a steep hill, had felt something give way and was seized with a sharp pain in chest, just over the third left costo-sternal articulation. Felt some shortness of breath, and after reaching home found that upon moving around or getting up she felt sick and heart palpitated. Having suffered from more or less palpitation of heart before, especially during her work (her occupation being to run a loom in a cotton mill), she paid but little attention to it, but it increasing and pain not subsiding she sent for me. Upon inspection in dorsal position, a slight pulsation visible at root of neck, just above the sternum. Upon sitting the pulsation disappeared and could not be seen anywhere. Palpation revealed a thrill and pulsation over upper portion of

*Read before the Southern Surgical and Gynecological Association.

thorax, most distinct in second right costal interspace, but felt over a space of three and a half or four inches in diameter and transmitted a short distance along the subclavian and carotid upon right side. Percussion gave slight dullness over upper part of sternum over a space of two inches in diameter, and extending from about one inch below the superior extremity of the sternum. Slight tenderness over this space but no softening; auscultation gave a well marked aneurismal bruit to be heard with the greatest distinctness in second right costal interspace close to sternum, but heard quite distinctly along the subclavian and carotids. Radial pulse not impaired, being apparently synchronous, some little laryngeal irritation. Pupil of right eye slightly contracted, and some little dimness of sight on that side. Drummond's sign of a systolic puffing upon stethoscopic auscultation over trachea proved of no value in this case. An apparent puffing was noted, but upon careful examination was found to be produced by the obscuring of the tracheal respiration by the loud bruit, thus giving an intermittency in respiration. It is possible, however, that in a large aneurism, and in a different position, more or less stenosis of the trachea might be produced, and in that way give rise to an apparent puffing. Diagnosed aneurism of the ascending portion of the aorta. Put her upon pot. iodide and tr. digitalis, the action of the heart being so tumultuous and irregular. Limited her diet and tried to follow Tiefnell's method. Upon October 28th, sent her to Mission Hospital. Upon more careful auscultation a mitral regurgitant and aortic stenotic murmur was heard, the first being transmitted over area common to aortic stenosis, there being a slight doubt as to differentiation between this murmur and the transmitted aneurismal bruit.

Upon October 29th, she spat some blood, but it was probably from a laryngeal or tracheal irritation. Commenced to complain of pain in back, deafness in right ear, and slight pain and difficulty in swallowing. The pulsation now is quite apparent at root of the neck, and can be felt along the carotids and subclavian for some distance. The action of the heart is regular now and she has suffered from no palpitation, but has had several attacks of asthma. The right pupil is contracted and the radial pulse upon the right side is getting a little weak and is a little later than the left. Unfortunately no sphymograph was convenient and so exactness could not be obtained. Tracings would have been of interest in this case.

Upon November 2d, finding the aneurism apparently growing, not at all affected by the iodide of potassium and dieting, I decided upon operation. By the way, I think many cases of cures by the iodide treatment are so many cases of mistaken diagnosis, many being reported as aneurisms the size of a walnut, pigeon's egg, etc. I imagine it would be very hard to diagnose such an aneurism, it not being likely that one of such small size would produce symptoms directing the attention of the patient to the aorta.

At the time of operation there was a dullness over some 3½ inches of diameter not well marked except over sternum. The radial pulse was quite perceptibly affected and involvement of the innominate artery was probable. Upon November 3d I operated at Mission Hospital, in this city, assisted by the staff. Chloroform was given, but she took it so badly, failure of respiration repeatedly recurring, that I substituted ether. I intended to tie the common carotid just above the omo-hyoid, but it being placed higher in the neck than usual,I came upon the carotid nearly half an inch below where it crosses. The vessel was exposed by drawing the sterno-mastoid outwardly and the sterno-hyoid inwardly, thus exposing the artery nicely. The descendens noni was just upon the artery, but was held aside while the ligature was passed from within out. The internal jugular was not seen. Catgut was used to ligate. The subclavian was tied in the third portion, just outside of the scalenus anticus. It was placed quite deep in its bed here and had to be raised some little distance before the ligature could be tied. The external jugular vein and a network of small veins crossed the middle of the incision, but by careful working none were cut. The jugular I dissected out and drew to the inner side of the incision. The brachial plexus was not seen nor was anything of importance except the artery. No blood was lost during the operation, the water hardly being discolored. The incisions were closed with very light catgut and iodoform and iodoform gauze used as a dressing. Antisepsis was strictly observed. Time of operation, one hour and a half.

Patient rallied well from operation, but suffered great nausea during the night. At 9 a. m. on November 4th, the patient was easy; no pain, but a slight heaviness in right arm, circulation good and well established, pulse in left arm a little increased, a distinct pulsation to be felt over abdominal aorto upon palpitation. Pulse 100. Temperature normal. On November 5th, some appetite and feeling comfortable. There was no rise of temperature during the entire period, and the pulse upon the third day went down to 80. Upon the fourth day after operation a small abscess formed at the root of the lower right canine tooth and ruptured, the tooth being decayed. Not a bad symptom appeared, no sign of aneurism, the only thing complained of being a little weakness in the right arm. All the subjective symptoms present before the operation had disappeared. The dressing was taken off on November 13th, the tenth day, and everything found united. No sign of pus, the dressing not even being stained. The pulsation had diminished somewhat, and from this time on got less and less. The patient got out of bed upon the twelfth day, and left the hospital on the seventeenth day after the operation. I put her on a syrup of the hypophosphites, and she gained strength daily. Upon the 27th of November, nineteen days after the operation, the pulsation could hardly be felt, and then only by deep and firm digital pressure upon the supra-sternal fossa. No thrill present upon the 30th day of November, the twenty-seventh day after the operation, no pulsation could be felt, but a slight systolic bruit could still be heard over the ascending aorta, being confused and lost in the aortic stenotic murmur. The abdominal pulsation had disappeared, and the only thing abnormal was a slight increase in the pulsation of the left carotid, there being a compensatory hypertrophy and dilation. Discharged as cured with directions to report every month or two, or upon anything abnormal happening.

The statistics of this operation are in some confusion, owing to several divisions made by different writers upon this subject. J.

A. Wyeth, of New York, claims to be the second to operate upon aneurism of the ascending aorta, this then being the third case of its kind, the first dying; Wyeth's, reported as a cure, dying in one year from diarrhœa, and my case, also a cure, being the second case. Gross, however, gives six cases, beginning with Bickersteth, in 1864, and ending with Palmer, in 1880, which, added to Wyeth's and the present case, make eight cases. For lack of time I have not collected statistics upon the operation, and will be thankful for any help in this direction which any member may give me.

This operation is practically in its infancy, the great mortality being due to delay in operation. So it has been with all operations, the results getting better as we operate earlier, it being, I think, a justifiable and practicable procedure. Very few patients are willing to go to bed for two or three years, conceding that the iodide of potash treatment is of service, and if taken in time hopes may be given of a cure, with but little risk, by this operation. It should not be undertaken by one faulty in his anatomy or in his surgery, and antisepsis must be thoroughly carried out.

